# Supplementary material for: Effectiveness and Safety of Ayurvedic Medicines in Type 2 Diabetes Mellitus Management: A Systematic Review and Meta-Analysis
Source: Front Pharmacol. 2022 Jun 8;13:821810. doi: 10.3389/fphar.2022.821810 (PMC9213670; doi:10.3389/fphar.2022.821810)
Supplement: Supplementary file 1 [file DataSheet1.zip › Supplementary Material/Forest and Funnel Plots/Portulaca oleracea/HDL-C.pdf]

Portulaca oleracea - HDL-C

	Intervention			Comparator			Mean Diff.	Weight
Study	N	Mean	SD	N	Mean	SD	with 95% CI	(%)
Farzanegi 2014	14	43.64	7.28	14	41.76	11.22	1.88 [-5.13, 8.89]	36.62
Dehghan 2016	16	42.95	19.93	16	36.70	26.75	6.25 [-10.10, 22.60]	6.84
Wainstein 2016	31	46.60	11.30	32	49.30	11.40	-2.70 [-8.31, 2.91]	56.54
**Overall**							-0.41 [-4.69, 3.87]	

Heterogeneity: $\tau^2 = 0.27$, $I^2 = 1.52\%$, $H^2 = 1.02$

Test of $\theta_i = \theta_j$: Q(2) = 1.69, p = 0.43

Test of $\theta = 0$: z = -0.19, p = 0.85

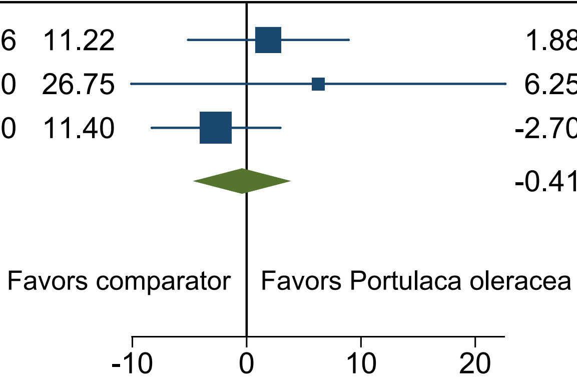

Random-effects REML model
